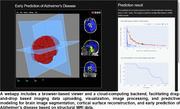# A web‐based tool for early prediction of Alzheimer’s disease dementia based on magnetic resonance imaging data

**DOI:** 10.1002/alz.093505

**Published:** 2025-01-09

**Authors:** Yong Fan

**Affiliations:** ^1^ Perelman School of Medicine, University of Pennsylvania, Philadelphia, PA USA

## Abstract

**Background:**

Recent studies have demonstrated that deep learning of magnetic resonance imaging (MRI) brain scans can accurately predict Alzheimer’s disease (AD) dementia and cognitive decline. However, the translational potential of this technique remains unfulfilled, as the underlying deep learning techniques are not yet available for immediate clinical use. To address this issue, we develop a web‐based tool to facilitate real‐time imaging data visualization and analyses, including brain image segmentation, cortical surface reconstruction, and early prediction of Alzheimer’s disease dementia based on structural MRI data.

**Method:**

A webapp has been developed to load and visualize imaging data with a browser‐based viewer and achieve fast imaging data processing and predictive modeling with a cloud‐computing backend. The browser‐based viewer provides a webpage‐based interface for communicating with the cloud‐computing backend and visualizing imaging data and analysis results. The cloud‐computing backend integrates state‐of‐the‐art deep learning algorithms to carry out real‐time brain image segmentation, cortical surface reconstruction, and early prediction of Alzheimer’s disease dementia based on structural MRI data. All the image processing and prediction results are automatically sent back to the browser‐based viewer for visualizing the results. This webapp supports imaging data in both DICOM and NIFTI formats and all identity information of imaging files are removed automatically before the imaging data being sent to the cloud‐computing backend for computing.

**Result:**

This webapp is compatible across widely used web browsers, allowing real‐time image analysis without installing any software. It can load and visualize MRI images with drag‐and‐drop and achieve brain image segmentation, cortical surface reconstruction, and prediction of progression of Alzheimer’s disease dementia and cognitive decline within seconds. Validation results on large imaging datasets, including ADNI and AIBL, have shown that this tool can achieve fast and accurate prediction of Alzheimer’s disease dementia and cognitive decline.

**Conclusion:**

The webapp turns deep learning methods for MRI data analysis into a clinically usable tool, illustrating the feasibility of real‐time predictive modeling of Alzheimer’s disease dementia based on MRI data. Ongoing development will further increase its capacity to facilitate predictive modeling of diverse neurodegenerative diseases based on imaging data.